# 2-Bromo-5,7-dimeth­oxy-4-phenyl­quinoline

**DOI:** 10.1107/S160053680900587X

**Published:** 2009-02-28

**Authors:** Veshal Gopal, Mohan Bhadbhade, Daniel Shiu Hin Chan, Chao-wei Leu, David StC Black, Naresh Kumar

**Affiliations:** a School of Chemistry, The University of New South Wales, Sydney, NSW 2052, Australia; b X-Ray Crystallography Laboratory, UNSW Analytical Centre, The University of New South Wales, Sydney, NSW 2052, Australia

## Abstract

The title compound, C_17_H_14_BrNO_2_, was synthesized by the treatment of 5,7-dimeth­oxy-4-phenyl­quinolin-2-one with phosphoryl bromide in a Vilsmeier-type reaction. There are two independent mol­ecules (*A* and *B*) in the asymmetric unit which differ by 11.2° in the orientation of the 4-phenyl ring with respect to the planar quinoline ring system [dihedral angles = 55.15 (8) and 66.34 (8)° in mol­ecules *A* and *B*, respectively]. In the crystal structure, the independent mol­ecules are linked *via* C—H⋯N and C—H⋯O hydrogen bonds, forming centrosymmetric tetra­meric units which are cross-linked through C—H⋯π and C—Br⋯π inter­actions with Br⋯centroid distances of 3.4289 (8) and 3.5967 (8) Å.

## Related literature

For a study of the anti­tumor activity of some 5,7-dimethoxy­quinolinlone analogues, see: Joseph *et al.* (2002 ▶).
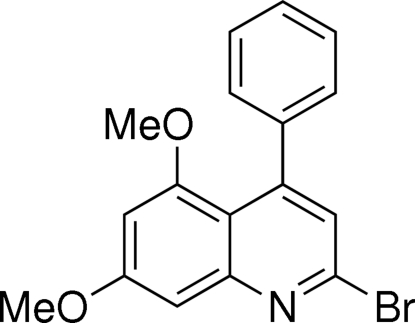


## Experimental

### 

#### Crystal data

C_17_H_14_BrNO_2_
*M* *_r_* = 344.20
Triclinic, 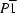

*a* = 9.7698 (2) Å
*b* = 9.9799 (3) Å
*c* = 14.8076 (4) Å
α = 93.499 (1)°
β = 95.154 (1)°
γ = 91.838 (1)°
*V* = 1434.22 (7) Å^3^
*Z* = 4
Mo *K*α radiation
μ = 2.87 mm^−1^
*T* = 150 K
0.39 × 0.19 × 0.18 mm

#### Data collection

Bruker Kappa APEXII CCD area-detector diffractometer
Absorption correction: multi-scan (*SADABS*; Sheldrick, 2003 ▶) *T*_min_ = 0.401, *T*_max_ = 0.626
27207 measured reflections
5008 independent reflections
4648 reflections with *I* > 2σ(*I*)
*R*_int_ = 0.060

#### Refinement

*R*[*F* ^2^ > 2σ(*F* ^2^)] = 0.029
*wR*(*F* ^2^) = 0.100
*S* = 0.87
5008 reflections
491 parameters
H atoms treated by a mixture of independent and constrained refinement
Δρ_max_ = 0.38 e Å^−3^
Δρ_min_ = −0.55 e Å^−3^

### 

Data collection: *APEX2* (Bruker, 2007 ▶); cell refinement: *SAINT* (Bruker, 2007 ▶); data reduction: *SAINT*; program(s) used to solve structure: *SHELXS97* (Sheldrick, 2008 ▶); program(s) used to refine structure: *SHELXL97* (Sheldrick, 2008 ▶); molecular graphics: *SHELXTL-Plus* (Sheldrick, 2008 ▶); software used to prepare material for publication: *SHELXL97*.

## Supplementary Material

Crystal structure: contains datablocks I, global. DOI: 10.1107/S160053680900587X/ci2767sup1.cif

Structure factors: contains datablocks I. DOI: 10.1107/S160053680900587X/ci2767Isup2.hkl

Additional supplementary materials:  crystallographic information; 3D view; checkCIF report

## Figures and Tables

**Table 1:** Hydrogen-bond geometry (Å, °)

*D*—H⋯*A*	*D*—H	H⋯*A*	*D*⋯*A*	*D*—H⋯*A*
C16*A*—H16*A*⋯N1*B*^i^	0.93 (2)	2.59 (2)	3.497 (3)	167 (2)
C16*B*—H16*B*⋯O2*A*^i^	0.92 (2)	2.54 (2)	3.437 (2)	163 (2)
C17*B*—H272⋯O2*B*^ii^	1.04 (3)	2.57 (3)	3.580 (3)	164 (2)
C12*A*—H12*A*⋯*Cg*1^iii^	0.95 (3)	2.87 (3)	3.762 (2)	158 (2)

Symmetry codes: (i) 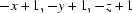; (ii) 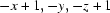; (iii) 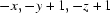. *Cg*1 is the centroid of the N1*B*/C2*B*–C4*B*/C9*B*/C10*B* ring.

**Fig. 1.**
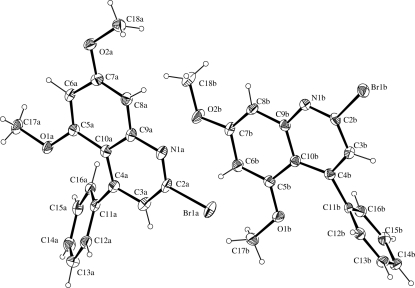
A view of two molecules in the asymmetric unit along with labelling of atoms. Displacement ellipsoids are drawn at the 50% probability level.

**Fig. 2.**
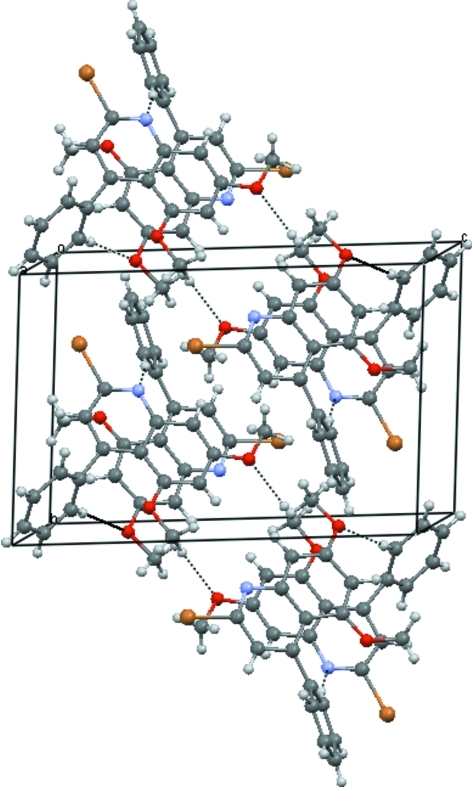
A view of the tetrameric unit formed by C—H···N and C—H···O hydrogen bonds (dashed lines).

## supplementary crystallographic information

### Comment

The title compound is a precursor to 2-arylquinolines, which are analogues of flavones such as chrysin. The consumption of flavones has been linked to lower incidences of hormone-dependent cancers, diabetes, obesity and cardiovascular diseases. The title compound was synthesized by the treatment of 5,7-dimethoxy-4-phenylquinolin-2-one with phosphoryl bromide.

The crystals contain two crystallographically independent molecules in the asymmetric unit (Fig. 1) which differ by 11.19 ° in the orientation of the 4-phenyl ring with respect to the planar quinoline moeity. The dihedral angle between the quinoline ring system and phenyl ring is 55.15 (8)° in molecule A and 66.34 (8)° in molecule B.

In the crystal structure, the two independent molecules are linked *via* C—H···N and C—H···O hydrogen bonds (Table 1) to form centrosymmetric tetrameric units (Fig. 2). The tetramers are cross-linked *via* C—H···π interactions (Table 1) involving the C12A—H12A group and the N1B/C2B—C4B/C9B/C10B ring. In addition, intermolecular C—Br···π interactions involving each independent molecule are observed between tetramers. The Br1A···*Cg*2 distance (*Cg*2 is the centroid of the C11A—C16A ring at -*x*, 1 - *y*, 1 - *z*) and C2A—Br1A···*Cg*2 angle are 3.5967 (8) Å and 135.59 (6)°, respectively, whereas, the Br1B···*Cg*3 distance (*Cg*3 is the centroid of the C11B—C16B ring at 1 - *x*, 1 - *y*, 2 - *z*) and C2B—Br1B···*Cg*3 angle are 3.4289 (8) Å and 149.71 (6) °, respectively.

### Experimental

To a solution of 5,7-dimethoxy-4-phenylquinolin-2-one (2.01 g, 7.1 mmol) in 1,2-dichloroethane (20 ml) was added dropwise a solution of phosphoryl bromide (6.32 g, 22.2 mmol) in 1,2-dichloroethane (20 ml) and the mixture was refluxed for 4 h. The crude product was purified by chromatography on silica gel (50% dichloromethane/hexane). Recrystallization from dichloromethane-hexane (2:3 *v*/*v*) afforded the title compound as light yellow needles (1.09 g, 44%).

### Refinement

All H atoms were located in a difference Fourier map and their positions and isotropic displacement parameters were refined freely [C—H = 0.87 (3) Å - 1.05 (3) Å].

### Crystal data

**Table:**

C_17_H_14_BrNO_2_	*Z* = 4
*M**_r_* = 344.20	*F*(000) = 696
Triclinic, *P*1	*D*_x_ = 1.594 Mg m^−^^3^
Hall symbol: -P 1	Mo *K*α radiation, λ = 0.71073 Å
*a* = 9.7698 (2) Å	Cell parameters from 9202 reflections
*b* = 9.9799 (3) Å	θ = 2.4–28.0°
*c* = 14.8076 (4) Å	µ = 2.87 mm^−^^1^
α = 93.499 (1)°	*T* = 150 K
β = 95.154 (1)°	Needle, light yellow
γ = 91.838 (1)°	0.39 × 0.19 × 0.18 mm
*V* = 1434.22 (7) Å^3^	

### Data collection

**Table:**

Bruker Kappa APEXII CCD area-detector diffractometer	5008 independent reflections
Radiation source: fine-focus sealed tube	4648 reflections with *I* > 2σ(*I*)
graphite	*R*_int_ = 0.060
φ scans, and ω scans with κ offsets	θ_max_ = 25.0°, θ_min_ = 1.4°
Absorption correction: multi-scan (*SADABS*; Sheldrick, 2003)	*h* = −11→11
*T*_min_ = 0.401, *T*_max_ = 0.626	*k* = −11→11
27207 measured reflections	*l* = −17→17

### Refinement

**Table:**

Refinement on *F*^2^	Primary atom site location: structure-invariant direct methods
Least-squares matrix: full	Secondary atom site location: difference Fourier map
*R*[*F*^2^ > 2σ(*F*^2^)] = 0.029	Hydrogen site location: inferred from neighbouring sites
*wR*(*F*^2^) = 0.100	H atoms treated by a mixture of independent and constrained refinement
*S* = 0.87	*w* = 1/[σ^2^(*F*_o_^2^) + (0.1*P*)^2^] where *P* = (*F*_o_^2^ + 2*F*_c_^2^)/3
5008 reflections	(Δ/σ)_max_ = 0.002
491 parameters	Δρ_max_ = 0.38 e Å^−^^3^
0 restraints	Δρ_min_ = −0.55 e Å^−^^3^

### Special details

**Table:**

Geometry. All e.s.d.'s (except the e.s.d. in the dihedral angle between two l.s. planes) are estimated using the full covariance matrix. The cell e.s.d.'s are taken into account individually in the estimation of e.s.d.'s in distances, angles and torsion angles; correlations between e.s.d.'s in cell parameters are only used when they are defined by crystal symmetry. An approximate (isotropic) treatment of cell e.s.d.'s is used for estimating e.s.d.'s involving l.s. planes.
Refinement. Refinement of *F*^2^ against ALL reflections. The weighted *R*-factor *wR* and goodness of fit *S* are based on *F*^2^, conventional *R*-factors *R* are based on *F*, with *F* set to zero for negative *F*^2^. The threshold expression of *F*^2^ > σ(*F*^2^) is used only for calculating *R*-factors(gt) *etc*. and is not relevant to the choice of reflections for refinement. *R*-factors based on *F*^2^ are statistically about twice as large as those based on *F*, and *R*- factors based on ALL data will be even larger.

### Fractional atomic coordinates and isotropic or equivalent isotropic displacement parameters (Å^2^)

**Table:**

	*x*	*y*	*z*	*U*_iso_*/*U*_eq_	
Br1A	−0.09830 (2)	0.72553 (2)	0.551387 (13)	0.02782 (11)	
N1A	0.06082 (17)	0.81444 (17)	0.42221 (11)	0.0205 (4)	
C2A	−0.0207 (2)	0.7146 (2)	0.43692 (13)	0.0202 (4)	
C3A	−0.0501 (2)	0.5984 (2)	0.38082 (14)	0.0223 (4)	
C4A	0.01131 (19)	0.5845 (2)	0.29994 (13)	0.0185 (4)	
C5A	0.15706 (19)	0.70379 (19)	0.19273 (13)	0.0176 (4)	
C6A	0.24532 (19)	0.8091 (2)	0.18021 (13)	0.0182 (4)	
C7A	0.27300 (19)	0.91362 (19)	0.24867 (13)	0.0197 (4)	
C8A	0.2096 (2)	0.9153 (2)	0.32701 (13)	0.0204 (4)	
C9A	0.11985 (19)	0.80637 (19)	0.34132 (13)	0.0186 (4)	
C10A	0.09499 (19)	0.69426 (19)	0.27681 (13)	0.0178 (4)	
C11A	−0.0078 (2)	0.4498 (2)	0.25100 (13)	0.0190 (4)	
C12A	−0.1389 (2)	0.3890 (2)	0.23443 (13)	0.0220 (4)	
C13A	−0.1566 (2)	0.2565 (2)	0.20000 (15)	0.0265 (5)	
C14A	−0.0440 (2)	0.1825 (2)	0.18294 (14)	0.0265 (5)	
C15A	0.0871 (2)	0.2423 (2)	0.19786 (13)	0.0236 (4)	
C16A	0.1057 (2)	0.3744 (2)	0.23107 (13)	0.0196 (4)	
C17A	0.1870 (2)	0.6020 (3)	0.04572 (15)	0.0267 (5)	
C18A	0.4127 (2)	1.1078 (2)	0.29986 (15)	0.0262 (5)	
O1A	0.11992 (14)	0.60380 (14)	0.12739 (9)	0.0216 (3)	
O2A	0.36495 (15)	1.01016 (14)	0.22783 (10)	0.0231 (3)	
Br1B	0.55112 (2)	0.694767 (19)	0.898615 (13)	0.02541 (11)	
N1B	0.54825 (17)	0.51735 (17)	0.74864 (11)	0.0187 (4)	
C2B	0.50047 (19)	0.53079 (19)	0.82786 (13)	0.0183 (4)	
C3B	0.4198 (2)	0.4360 (2)	0.86636 (14)	0.0191 (4)	
C4B	0.38014 (19)	0.3180 (2)	0.81667 (13)	0.0169 (4)	
C5B	0.37631 (19)	0.19046 (19)	0.66232 (13)	0.0183 (4)	
C6B	0.4281 (2)	0.1790 (2)	0.57944 (14)	0.0205 (4)	
C7B	0.5251 (2)	0.2767 (2)	0.55562 (13)	0.0200 (4)	
C8B	0.5642 (2)	0.3868 (2)	0.61213 (14)	0.0193 (4)	
C9B	0.51012 (19)	0.40097 (19)	0.69749 (13)	0.0175 (4)	
C10B	0.42110 (19)	0.30043 (19)	0.72675 (13)	0.0173 (4)	
C11B	0.30626 (19)	0.2160 (2)	0.86651 (12)	0.0161 (4)	
C12B	0.1834 (2)	0.2501 (2)	0.90227 (13)	0.0185 (4)	
C13B	0.1267 (2)	0.1686 (2)	0.96236 (14)	0.0223 (4)	
C14B	0.1912 (2)	0.0542 (2)	0.98908 (14)	0.0228 (4)	
C15B	0.3122 (2)	0.0188 (2)	0.95183 (14)	0.0218 (4)	
C16B	0.36828 (19)	0.0984 (2)	0.89061 (13)	0.0192 (4)	
C17B	0.2224 (2)	0.0005 (2)	0.62431 (15)	0.0236 (4)	
C18B	0.6704 (2)	0.3438 (2)	0.44447 (16)	0.0266 (5)	
O1B	0.28035 (15)	0.10469 (14)	0.68888 (9)	0.0238 (3)	
O2B	0.57054 (16)	0.25043 (15)	0.47225 (9)	0.0276 (3)	
H3A	−0.102 (3)	0.527 (3)	0.3972 (17)	0.030 (7)*	
H6A	0.290 (2)	0.816 (2)	0.1237 (16)	0.020 (5)*	
H8A	0.220 (2)	0.987 (3)	0.3705 (16)	0.026 (6)*	
H16A	0.193 (2)	0.414 (2)	0.2432 (15)	0.021 (6)*	
H14A	−0.053 (2)	0.098 (3)	0.1641 (16)	0.027 (6)*	
H13A	−0.241 (3)	0.218 (3)	0.1921 (19)	0.038 (7)*	
H15A	0.163 (3)	0.187 (3)	0.1838 (16)	0.033 (6)*	
H3B	0.394 (2)	0.452 (2)	0.9274 (17)	0.027 (6)*	
H8B	0.622 (2)	0.455 (2)	0.5995 (15)	0.021 (6)*	
H12B	0.140 (2)	0.327 (2)	0.8887 (13)	0.010 (5)*	
H13B	0.051 (3)	0.194 (3)	0.9858 (18)	0.038 (7)*	
H16B	0.451 (2)	0.079 (2)	0.8690 (15)	0.020 (5)*	
H15B	0.363 (2)	−0.057 (3)	0.9700 (16)	0.026 (6)*	
H6B	0.406 (2)	0.113 (2)	0.5367 (17)	0.024 (6)*	
H12A	−0.217 (3)	0.436 (2)	0.2490 (15)	0.029 (6)*	
H181	0.480 (3)	1.150 (3)	0.2743 (19)	0.038 (7)*	
H182	0.340 (3)	1.171 (3)	0.3149 (17)	0.033 (7)*	
H183	0.444 (2)	1.058 (2)	0.3530 (17)	0.027 (6)*	
H171	0.163 (2)	0.683 (2)	0.0128 (16)	0.022 (5)*	
H172	0.152 (3)	0.531 (3)	0.012 (2)	0.042 (7)*	
H173	0.283 (3)	0.593 (3)	0.0583 (17)	0.028 (6)*	
H282	0.748 (3)	0.348 (3)	0.4829 (18)	0.032 (7)*	
H281	0.634 (2)	0.429 (3)	0.4422 (15)	0.023 (6)*	
H283	0.688 (3)	0.308 (3)	0.3863 (19)	0.032 (7)*	
H273	0.156 (3)	−0.042 (3)	0.6514 (19)	0.043 (8)*	
H272	0.298 (3)	−0.065 (3)	0.6064 (16)	0.034 (6)*	
H271	0.177 (2)	0.035 (2)	0.5686 (17)	0.027 (6)*	
H14B	0.151 (2)	0.000 (2)	1.0286 (16)	0.022 (6)*	

### Atomic displacement parameters (Å^2^)

**Table:**

	*U*^11^	*U*^22^	*U*^33^	*U*^12^	*U*^13^	*U*^23^
Br1A	0.03787 (17)	0.02967 (17)	0.01893 (16)	0.00803 (11)	0.01470 (11)	0.00473 (11)
N1A	0.0251 (9)	0.0211 (9)	0.0169 (8)	0.0059 (7)	0.0071 (7)	0.0031 (7)
C2A	0.0236 (10)	0.0240 (11)	0.0147 (9)	0.0077 (8)	0.0072 (8)	0.0052 (8)
C3A	0.0243 (10)	0.0216 (11)	0.0231 (11)	0.0050 (8)	0.0082 (8)	0.0065 (9)
C4A	0.0174 (9)	0.0207 (10)	0.0183 (10)	0.0042 (7)	0.0030 (7)	0.0039 (8)
C5A	0.0183 (9)	0.0176 (10)	0.0175 (9)	0.0043 (7)	0.0037 (7)	0.0011 (8)
C6A	0.0209 (9)	0.0200 (10)	0.0150 (10)	0.0042 (8)	0.0056 (8)	0.0034 (8)
C7A	0.0202 (9)	0.0173 (9)	0.0223 (10)	0.0031 (7)	0.0022 (8)	0.0047 (8)
C8A	0.0244 (10)	0.0185 (10)	0.0183 (10)	0.0025 (8)	0.0038 (8)	−0.0015 (8)
C9A	0.0211 (9)	0.0199 (10)	0.0157 (10)	0.0074 (8)	0.0035 (7)	0.0037 (8)
C10A	0.0184 (9)	0.0188 (10)	0.0171 (10)	0.0055 (7)	0.0021 (7)	0.0034 (8)
C11A	0.0224 (10)	0.0219 (11)	0.0137 (10)	0.0018 (8)	0.0039 (8)	0.0047 (8)
C12A	0.0190 (10)	0.0286 (11)	0.0192 (10)	0.0026 (9)	0.0026 (8)	0.0062 (9)
C13A	0.0233 (11)	0.0311 (12)	0.0242 (11)	−0.0072 (9)	−0.0001 (9)	0.0030 (9)
C14A	0.0390 (12)	0.0202 (12)	0.0192 (11)	−0.0047 (9)	0.0023 (9)	−0.0030 (9)
C15A	0.0283 (11)	0.0273 (11)	0.0156 (10)	0.0055 (9)	0.0042 (8)	−0.0002 (8)
C16A	0.0187 (10)	0.0246 (11)	0.0159 (10)	0.0008 (8)	0.0035 (8)	0.0028 (8)
C17A	0.0328 (13)	0.0314 (13)	0.0165 (10)	0.0019 (10)	0.0095 (9)	−0.0040 (9)
C18A	0.0297 (11)	0.0227 (11)	0.0252 (12)	−0.0050 (9)	−0.0003 (9)	0.0021 (9)
O1A	0.0274 (7)	0.0233 (7)	0.0146 (7)	−0.0021 (6)	0.0081 (5)	−0.0027 (6)
O2A	0.0268 (7)	0.0205 (7)	0.0226 (8)	−0.0052 (6)	0.0074 (6)	0.0002 (6)
Br1B	0.03604 (17)	0.01914 (16)	0.01965 (16)	−0.00615 (10)	−0.00020 (10)	−0.00227 (10)
N1B	0.0190 (8)	0.0197 (9)	0.0170 (8)	−0.0013 (6)	0.0007 (6)	0.0009 (7)
C2B	0.0220 (9)	0.0152 (9)	0.0165 (10)	0.0003 (7)	−0.0026 (8)	−0.0009 (7)
C3B	0.0219 (10)	0.0200 (10)	0.0157 (10)	0.0011 (8)	0.0026 (8)	0.0021 (8)
C4B	0.0147 (9)	0.0182 (10)	0.0176 (10)	0.0021 (7)	0.0004 (7)	0.0012 (8)
C5B	0.0190 (9)	0.0167 (10)	0.0191 (10)	0.0010 (7)	0.0000 (7)	0.0032 (8)
C6B	0.0237 (10)	0.0191 (10)	0.0183 (10)	0.0003 (8)	0.0031 (8)	−0.0027 (8)
C7B	0.0236 (10)	0.0236 (11)	0.0140 (10)	0.0046 (8)	0.0053 (8)	0.0031 (8)
C8B	0.0202 (10)	0.0204 (10)	0.0185 (10)	−0.0003 (8)	0.0048 (8)	0.0059 (8)
C9B	0.0169 (9)	0.0179 (10)	0.0176 (9)	0.0012 (7)	−0.0011 (7)	0.0028 (8)
C10B	0.0170 (9)	0.0196 (10)	0.0157 (9)	0.0028 (7)	0.0011 (7)	0.0033 (8)
C11B	0.0183 (9)	0.0190 (10)	0.0105 (9)	−0.0033 (7)	0.0009 (7)	−0.0017 (7)
C12B	0.0188 (9)	0.0184 (11)	0.0179 (10)	0.0019 (8)	0.0013 (8)	−0.0023 (8)
C13B	0.0180 (10)	0.0284 (11)	0.0208 (10)	−0.0014 (8)	0.0071 (8)	−0.0035 (8)
C14B	0.0274 (10)	0.0226 (11)	0.0182 (10)	−0.0087 (8)	0.0057 (8)	0.0003 (8)
C15B	0.0255 (10)	0.0180 (10)	0.0216 (10)	0.0010 (8)	0.0012 (8)	0.0007 (8)
C16B	0.0180 (10)	0.0205 (10)	0.0189 (10)	−0.0003 (8)	0.0046 (8)	−0.0032 (8)
C17B	0.0263 (11)	0.0195 (11)	0.0238 (11)	−0.0040 (9)	0.0018 (9)	−0.0044 (9)
C18B	0.0319 (12)	0.0260 (12)	0.0246 (12)	0.0037 (10)	0.0134 (10)	0.0073 (9)
O1B	0.0283 (7)	0.0234 (7)	0.0192 (7)	−0.0094 (6)	0.0055 (6)	−0.0020 (6)
O2B	0.0366 (8)	0.0284 (8)	0.0195 (8)	−0.0021 (6)	0.0152 (6)	−0.0023 (6)

### Geometric parameters (Å, °)

**Table:**

Br1A—C2A	1.9168 (19)	Br1B—C2B	1.9147 (19)
N1A—C2A	1.296 (3)	N1B—C2B	1.302 (3)
N1A—C9A	1.374 (3)	N1B—C9B	1.370 (3)
C2A—C3A	1.392 (3)	C2B—C3B	1.393 (3)
C3A—C4A	1.388 (3)	C3B—C4B	1.376 (3)
C3A—H3A	0.92 (3)	C3B—H3B	0.97 (3)
C4A—C10A	1.422 (3)	C4B—C10B	1.427 (3)
C4A—C11A	1.487 (3)	C4B—C11B	1.497 (3)
C5A—O1A	1.364 (2)	C5B—O1B	1.350 (2)
C5A—C6A	1.370 (3)	C5B—C6B	1.369 (3)
C5A—C10A	1.440 (3)	C5B—C10B	1.440 (3)
C6A—C7A	1.412 (3)	C6B—C7B	1.419 (3)
C6A—H6A	0.98 (2)	C6B—H6B	0.89 (3)
C7A—C8A	1.363 (3)	C7B—O2B	1.362 (2)
C7A—O2A	1.364 (2)	C7B—C8B	1.363 (3)
C8A—C9A	1.413 (3)	C8B—C9B	1.414 (3)
C8A—H8A	0.93 (3)	C8B—H8B	0.91 (2)
C9A—C10A	1.426 (3)	C9B—C10B	1.420 (3)
C11A—C12A	1.395 (3)	C11B—C16B	1.389 (3)
C11A—C16A	1.402 (3)	C11B—C12B	1.399 (3)
C12A—C13A	1.387 (3)	C12B—C13B	1.382 (3)
C12A—H12A	0.95 (3)	C12B—H12B	0.92 (2)
C13A—C14A	1.379 (3)	C13B—C14B	1.383 (3)
C13A—H13A	0.89 (3)	C13B—H13B	0.89 (3)
C14A—C15A	1.389 (3)	C14B—C15B	1.395 (3)
C14A—H14A	0.87 (3)	C14B—H14B	0.92 (2)
C15A—C16A	1.379 (3)	C15B—C16B	1.380 (3)
C15A—H15A	0.97 (3)	C15B—H15B	0.96 (3)
C16A—H16A	0.93 (2)	C16B—H16B	0.92 (2)
C17A—O1A	1.426 (2)	C17B—O1B	1.435 (2)
C17A—H171	0.99 (2)	C17B—H273	0.90 (3)
C17A—H172	0.88 (3)	C17B—H272	1.05 (3)
C17A—H173	0.95 (3)	C17B—H271	0.99 (2)
C18A—O2A	1.435 (3)	C18B—O2B	1.432 (3)
C18A—H181	0.90 (3)	C18B—H282	0.91 (3)
C18A—H182	1.00 (3)	C18B—H281	0.94 (3)
C18A—H183	0.99 (3)	C18B—H283	0.95 (3)
			
C2A—N1A—C9A	116.40 (17)	C2B—N1B—C9B	116.28 (17)
N1A—C2A—C3A	126.61 (18)	N1B—C2B—C3B	126.12 (18)
N1A—C2A—Br1A	115.79 (15)	N1B—C2B—Br1B	116.34 (14)
C3A—C2A—Br1A	117.47 (15)	C3B—C2B—Br1B	117.52 (14)
C4A—C3A—C2A	118.63 (19)	C4B—C3B—C2B	118.86 (18)
C4A—C3A—H3A	118.2 (16)	C4B—C3B—H3B	120.4 (15)
C2A—C3A—H3A	123.0 (16)	C2B—C3B—H3B	120.7 (15)
C3A—C4A—C10A	117.62 (18)	C3B—C4B—C10B	117.97 (18)
C3A—C4A—C11A	115.29 (18)	C3B—C4B—C11B	115.31 (17)
C10A—C4A—C11A	126.82 (17)	C10B—C4B—C11B	126.54 (17)
O1A—C5A—C6A	123.19 (17)	O1B—C5B—C6B	123.94 (18)
O1A—C5A—C10A	115.72 (16)	O1B—C5B—C10B	115.64 (16)
C6A—C5A—C10A	121.07 (18)	C6B—C5B—C10B	120.40 (18)
C5A—C6A—C7A	120.33 (17)	C5B—C6B—C7B	120.30 (19)
C5A—C6A—H6A	122.2 (13)	C5B—C6B—H6B	125.0 (15)
C7A—C6A—H6A	117.5 (13)	C7B—C6B—H6B	114.7 (15)
C8A—C7A—O2A	124.71 (18)	O2B—C7B—C8B	124.74 (19)
C8A—C7A—C6A	121.31 (18)	O2B—C7B—C6B	113.86 (18)
O2A—C7A—C6A	113.98 (16)	C8B—C7B—C6B	121.38 (19)
C7A—C8A—C9A	118.89 (18)	C7B—C8B—C9B	118.94 (19)
C7A—C8A—H8A	122.6 (14)	C7B—C8B—H8B	125.6 (15)
C9A—C8A—H8A	118.5 (14)	C9B—C8B—H8B	115.4 (15)
N1A—C9A—C8A	115.57 (17)	N1B—C9B—C8B	115.72 (17)
N1A—C9A—C10A	122.43 (17)	N1B—C9B—C10B	122.91 (17)
C8A—C9A—C10A	121.98 (17)	C8B—C9B—C10B	121.37 (17)
C4A—C10A—C9A	118.10 (17)	C9B—C10B—C4B	117.57 (17)
C4A—C10A—C5A	125.79 (18)	C9B—C10B—C5B	117.28 (17)
C9A—C10A—C5A	116.11 (17)	C4B—C10B—C5B	125.13 (18)
C12A—C11A—C16A	118.49 (19)	C16B—C11B—C12B	119.27 (18)
C12A—C11A—C4A	120.08 (18)	C16B—C11B—C4B	120.99 (17)
C16A—C11A—C4A	120.93 (17)	C12B—C11B—C4B	118.87 (17)
C13A—C12A—C11A	120.8 (2)	C13B—C12B—C11B	119.92 (19)
C13A—C12A—H12A	118.8 (14)	C13B—C12B—H12B	118.0 (13)
C11A—C12A—H12A	120.3 (14)	C11B—C12B—H12B	122.0 (13)
C14A—C13A—C12A	120.2 (2)	C12B—C13B—C14B	120.80 (19)
C14A—C13A—H13A	120.2 (18)	C12B—C13B—H13B	118.1 (18)
C12A—C13A—H13A	119.6 (18)	C14B—C13B—H13B	121.0 (18)
C13A—C14A—C15A	119.7 (2)	C13B—C14B—C15B	119.15 (19)
C13A—C14A—H14A	121.3 (15)	C13B—C14B—H14B	119.4 (14)
C15A—C14A—H14A	119.1 (15)	C15B—C14B—H14B	121.4 (14)
C16A—C15A—C14A	120.6 (2)	C16B—C15B—C14B	120.41 (19)
C16A—C15A—H15A	122.8 (15)	C16B—C15B—H15B	117.0 (14)
C14A—C15A—H15A	116.6 (15)	C14B—C15B—H15B	122.5 (14)
C15A—C16A—C11A	120.26 (19)	C15B—C16B—C11B	120.38 (18)
C15A—C16A—H16A	121.2 (14)	C15B—C16B—H16B	120.7 (14)
C11A—C16A—H16A	118.4 (14)	C11B—C16B—H16B	118.7 (14)
O1A—C17A—H171	109.2 (13)	O1B—C17B—H273	106.0 (19)
O1A—C17A—H172	106.1 (18)	O1B—C17B—H272	110.5 (13)
H171—C17A—H172	108 (2)	H273—C17B—H272	111 (2)
O1A—C17A—H173	110.7 (15)	O1B—C17B—H271	113.2 (14)
H171—C17A—H173	114 (2)	H273—C17B—H271	107 (2)
H172—C17A—H173	109 (2)	H272—C17B—H271	108.9 (19)
O2A—C18A—H181	100.4 (18)	O2B—C18B—H282	111.3 (16)
O2A—C18A—H182	112.2 (15)	O2B—C18B—H281	110.0 (14)
H181—C18A—H182	111 (2)	H282—C18B—H281	109 (2)
O2A—C18A—H183	107.3 (14)	O2B—C18B—H283	103.4 (16)
H181—C18A—H183	114 (2)	H282—C18B—H283	110 (2)
H182—C18A—H183	111 (2)	H281—C18B—H283	112 (2)
C5A—O1A—C17A	118.03 (16)	C5B—O1B—C17B	118.11 (16)
C7A—O2A—C18A	116.35 (16)	C7B—O2B—C18B	116.83 (17)

### Hydrogen-bond geometry (Å, °)

**Table:**

*D*—H···*A*	*D*—H	H···*A*	*D*···*A*	*D*—H···*A*
C16A—H16A···N1B^i^	0.93 (2)	2.59 (2)	3.497 (3)	167 (2)
C16B—H16B···O2A^i^	0.92 (2)	2.54 (2)	3.437 (2)	163 (2)
C17B—H272···O2B^ii^	1.04 (3)	2.57 (3)	3.580 (3)	164 (2)
C12A—H12A···Cg1^iii^	0.95 (3)	2.87 (3)	3.762 (2)	158 (2)

Symmetry codes: (i) −*x*+1, −*y*+1, −*z*+1; (ii) −*x*+1, −*y*, −*z*+1; (iii) −*x*, −*y*+1, −*z*+1.
